# Oridonin ameliorates carbon tetrachloride‐induced liver fibrosis in mice through inhibition of the NLRP3 inflammasome

**DOI:** 10.1002/ddr.21649

**Published:** 2020-03-26

**Authors:** Dong Liu, Hailong Qin, Bixian Yang, Bin Du, Xuelin Yun

**Affiliations:** ^1^ College of Pharmacy Guizhou University of Traditional Chinese Medicine Guiyang Guizhou China; ^2^ College of Food and Pharmacy Engineering Guiyang University Guiyang Guizhou China

**Keywords:** collagen deposition, HSCs activation, liver fibrosis, NLRP3, oridonin

## Abstract

Liver fibrosis is characterized by the activation of hepatic stellate cells (HSCs) and accumulation of the extracellular matrix. There are limitations in the current therapies for liver fibrosis. Recently, oridonin was shown to induce apoptosis in HSCs. Thus, we aimed to determine the roles of oridonin in chronic liver injury and fibrosis. Liver fibrosis was induced by CCl_4_ in mice injected intraperitoneally with oridonin for 6 weeks. The administration of oridonin significantly attenuated liver injury and reduced ALT levels. In addition, Sirius Red staining and the expression of α‐smooth muscle actin (α‐SMA) were significantly reduced by oridonin in murine livers with fibrosis. The expression of NLRP3, caspase‐1, and IL‐1β was downregulated with the oridonin treatment. Furthermore, the expression of F4/80 in liver tissues was also decreased by oridonin treatment. These results demonstrate that oridonin ameliorates chronic liver injury and fibrosis. Mechanically, oridonin may inhibit the activity of the NLRP3 inflammasome and inflammation in the liver. These results highlight the potential of oridonin as a therapeutic agent for liver fibrosis.

**Abbreviations**
ALT: alanine aminotransferase
CCl_4_: carbon tetrachloride
ECM: extracellular matrix
H&E: hematoxylin–eosin
HRP: horseradish peroxidase
HSCs: hepatic stellate cells
PVDF: polyvinylidene difluoride.
TGF‐β: transforming growth factor
α‐SMA: α‐smooth muscle actin

## 1 INTRODUCTION

Liver fibrosis is the pathological degradation of liver structure and function because of repeated damage and inflammation. It is also one of the stages involved in the development of various chronic liver diseases and cirrhosis (Lee & Friedman, 2011). This intractable disease is caused by the accumulation of extracellular matrix (ECM) proteins in the process of chronic liver injury. Hence, one of the vital steps in the treatment of liver fibrosis is to prevent the excessive deposition of ECM (Bataller & Brenner, 2005). Activated hepatic stellate cells (HSCs) are the main cells that synthesize ECM in the liver. The inhibition of their activation is thus potentially an effective therapy (Chen et al., 2018; Lan et al., 2018). Since the activation of HSCs is stimulated by transforming growth factor‐β (TGF‐β) and characterized by the expression of α‐smooth muscle actin (α‐SMA), the detection of this cytokine is necessary for the in vitro evaluation of liver fibrosis therapies (X. F. Wang et al., 2014). Anti‐inflammatory therapy is also a potential method to control liver fibrosis, as inflammation is present in various liver fibrosis rodent models (Alegre, Pelegrin, & Feldstein, 2017). Liver macrophages, also known as Kupffer cells, play a central role in liver inflammation. They interact with HSCs to modulate their phenotype (Koyama & Brenner, 2017). HSCs can also be activated directly by several damage‐associated molecular patterns (DAMPs) to produce a pro‐inflammatory effect. This is especially in NLRP3 inflammasome‐mediated inflammatory responses, which play a key role in the development of chronic liver disease and fibrosis (Inzaugarat et al., 2019; H. Wang, Liu, Wang, Chang, & Wang, 2016). Although many studies have attempted to elucidate the pathogenesis of liver fibrosis, there are still no approved drugs (Lin et al., 2018); thus, there is an urgent need to find an effective drug for the treatment of this disease (X. Zhao, Rui, et al., 2017).

**Figure 1:**
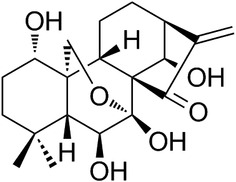
The structure of oridonin

Several studies have demonstrated that oridonin can potentially be used to treat liver fibrosis by inducing apoptosis in HSCs (Kuo et al., 2014). Oridonin is the main active ingredient in an herb named *Rabdosia rubescens*. This herb has been found to exhibit various medical effects, such as anti‐cancer, antibacterial, and anti‐inflammatory effects (Bohanon et al., 2014; Ding et al., 2016; He et al., 2018; J. Xu, Wold, Ding, Shen, & Zhou, 2018). As a natural compound extracted from *Rabdosia rubescens*, oridonin has been increasingly gaining attention (Figure 1). Bohanon et al. demonstrated the ability of oridonin and its analogs to increase apoptosis and decrease ECM production in HSCs (Bohanon et al., 2015). Meanwhile, data have shown that oridonin and its analogs have a remarkable effect on the inhibition of HSC proliferation (Bohanon, Wang, Graham, Ding, et al., 2015; Bohanon, Wang, Graham, Prasai, et al., 2015). However, the effects of oridonin in vivo remain unclear. In addition, increasing evidence has suggested that oridonin and its analogs may play a vital role in inhibiting inflammatory responses in HSCs, which allows their continued activation (Cummins, Wang, Sommerhalder, et al., 2018; Cummins, Wang, Xu, et al., 2018). Recently, it was confirmed that oridonin could bind NLRP3 inflammasome covalently and act as an NLRP3 inhibitor (He et al., 2018). Taken together, we hypothesize that oridonin attenuates liver fibrosis in vitro via the inhibition of the NLRP3 inflammasome.

In this study, we show that oridonin markedly attenuates CCl_4_‐induced liver fibrosis in mice and reduced the infiltration of inflammatory cells such as Kupffer cells. More importantly, the targeting of NLRP3 inflammasome in HSCs by oridonin contributes to HSC biology and fibrogenesis. Therefore, oridonin is a potential therapeutic agent for liver fibrosis.

## 2 MATERIALS AND METHODS

### 2.1 Materials and reagents

Oridonin was obtained from Sigma‐Aldrich [Purity 98.0% (HPLC), St. Louis, MO]. Carbon tetrachloride (CCl_4_) and corn oil were purchased from Aladdin (Shanghai, China). Antibodies against α‐SMA and β‐actin were from BOSTER (Wuhan, China) and TransGen (Beijing, China), respectively; horseradish peroxidase (HRP)‐conjugated Affinity Pure goat anti‐mouse IgG and anti‐rabbit IgG were purchased from Zhongshan Golden Bridge Biotechnology Co, Ltd. (Beijing, China). Enhanced chemiluminescence detection reagents were purchased from Clinx (Shanghai, China) and the chemiluminescence system was obtained from New Life Science Products (Boston, MA). Alanine aminotransferase (ALT) and hydroxyproline assay kits were purchased from Nanjing Jiancheng Bioengineering Institute (Nanjing, China).

### 2.2 Animals and treatment

All animal care and animal experiments were conducted in accordance with the China Animal Legislation and were approved by the Ethics Committee on the Care and Use of Laboratory Animals in Guangdong Pharmaceutical University (Guangzhou, China). Eight‐week‐old C57BL/6J mice (22–25 g) were purchased from the Experimental Animals Center of Guangdong Province, China and were divided into three groups (*n* = 5/group). Mice from the liver fibrosis group and the treatment group were given an intraperitoneal (IP) injection of CCl_4_ (diluted in corn oil at 1:4, 5 ml/kg body weight) twice a week (every Monday and Thursday) for 6 weeks. Injection with corn oil and saline was used as a control. Intraperitoneal injection of oridonin (dissolved in saline at the dose of 5 mg/kg) was performed daily, except Monday and Thursday, in the treatment group for 6 weeks as well. Saline was injected as a control reagent for oridonin. All mice were narcotized using ether after the final injection to get the blood sample through the orbital vein. After the mice were finally euthanized by cervical dislocation, liver specimens were harvested and stored at −80°C for further assays.

### 2.3 H&E and Sirius red staining

Liver specimens fixed in 10% buffered formalin were embedded in paraffin blocks and cut into slices (3 μm thick). Liver slices were then stained with hematoxylin–eosin (H&E) for routine histology and Sirius Red for collagen fiber. The images obtained from H&E and Sirius Red staining were captured by Olympus BX43 imaging.

### 2.4 Serum biochemistry

Standard enzymatic procedures were conducted according to the manufacturer's instruction (Nanjing Jiancheng Bioengineering Institute, Nanjing, China) to measure the serum levels of ALT.

### 2.5 Hydroxyproline assay

The hepatic hydroxyproline content was quantified by a colorimetric assay using a commercial kit (Nanjing Jiancheng Bioengineering Institute, Nanjing, China).

### 2.6 Immunohistochemistry and immunofluorescent staining

After routine deparaffinization, hydration, and blockage of endogenous peroxidase, sections were pretreated by microwaving for 20 min in 10 mmol/L sodium citrate buffer (pH 6.0) for antigen retrieval. This was followed by incubation sequentially with a blocking agent, α‐SMA antibodies (1:100, Boster, China), and a secondary antibody (1:200, Promega, Madison, WI). Slides were incubated with diaminobenzidine, followed with a brief counterstaining with hematoxylin, and mounted with Vectashield (Vector Labs, Burlingame, CA). The areas‐of‐interest were photographed and converted to a digital image using a light microscope equipped with a camera (Olympus BX51, NY). For immunofluorescence staining, liver specimens were fixed in 10% buffered formalin and sequentially exposed to 10 and 30% sucrose in PBS for 10 hr each and then embedded in Tissue Tek OTC compound (Sakura Finetek, Torrance, CA). The liver sections were permeabilized using 0.25% Triton X‐100 and incubated with a primary antibody against F4/80 (1:200, Abcam, Cambridge, MA). The liver sections were then incubated with the corresponding Alexa Fluor 594‐conjugated secondary antibodies (1:200, Invitrogen, Carlsbad, CA) for 1 hr at room temperature and stained with DAPI (1 μg/ml) for 10 min. Finally, the stained sections were viewed and photographed using a confocal microscope (Leica TCS SP5, Leica, Mannheim, Germany).

### 2.7 Cell culture and treatment

LX‐2 cells were maintained in DMEM supplemented with 10% FCS, 50 IU/ml penicillin, 50 μg/ml streptomycin, 2 mM l‐glutamine, and 1 mM sodium pyruvate at 37°C in a humidified 5% CO2 incubator. LX‐2 cells were seeded at 3 × 10^4^ per cm^2^ and grown in DMEM supplemented with 10% FCS until confluence was reached. LX‐2 cells were incubated with oridonin (1.25 μM) for 24 hr and then treated with TGF‐β1 (2 ng/ml) for 18 hr. Protein and RNA were then harvested for further assays.

### 2.8 Western blot analysis

Total proteins of the liver were extracted using RIPA lysis buffer and electrophoresed on a 10% SDS‐PAGE gel before transfer onto polyvinylidene difluoride (PVDF) membranes (Millipore Corp., Bedford, MA). The membranes were blocked with 5% nonfat milk in Tris‐buffered saline with Tween‐20 for 1 hr at room temperature and then incubated with antibodies against α‐SMA (1:5,000, BOSTER, Wuhan, China) and β‐actin (1:5,000, TransGen, Beijing, China) at 4°C overnight. The membranes were then incubated with their respective secondary antibodies (1:5,000) for 1 hr at room temperature. The bands were detected using enhanced chemiluminescence detection reagents (Clinx, Shanghai, China) and captured using a chemiluminescence system (New Life Science Products, Boston, MA).

### 2.9 Statistical analysis

All experiments were performed in at least triplicate. Values are expressed as mean ± *SD*. The differences between multiple groups of data were analyzed using one‐way ANOVA with Bonferroni correction (GraphPad Prism 7.0). *p* < .05 was considered statistically significant.

## 3 RESULTS

### 3.1 Oridonin attenuates CCl_4_‐induced liver injury in mice

**Figure 2:**
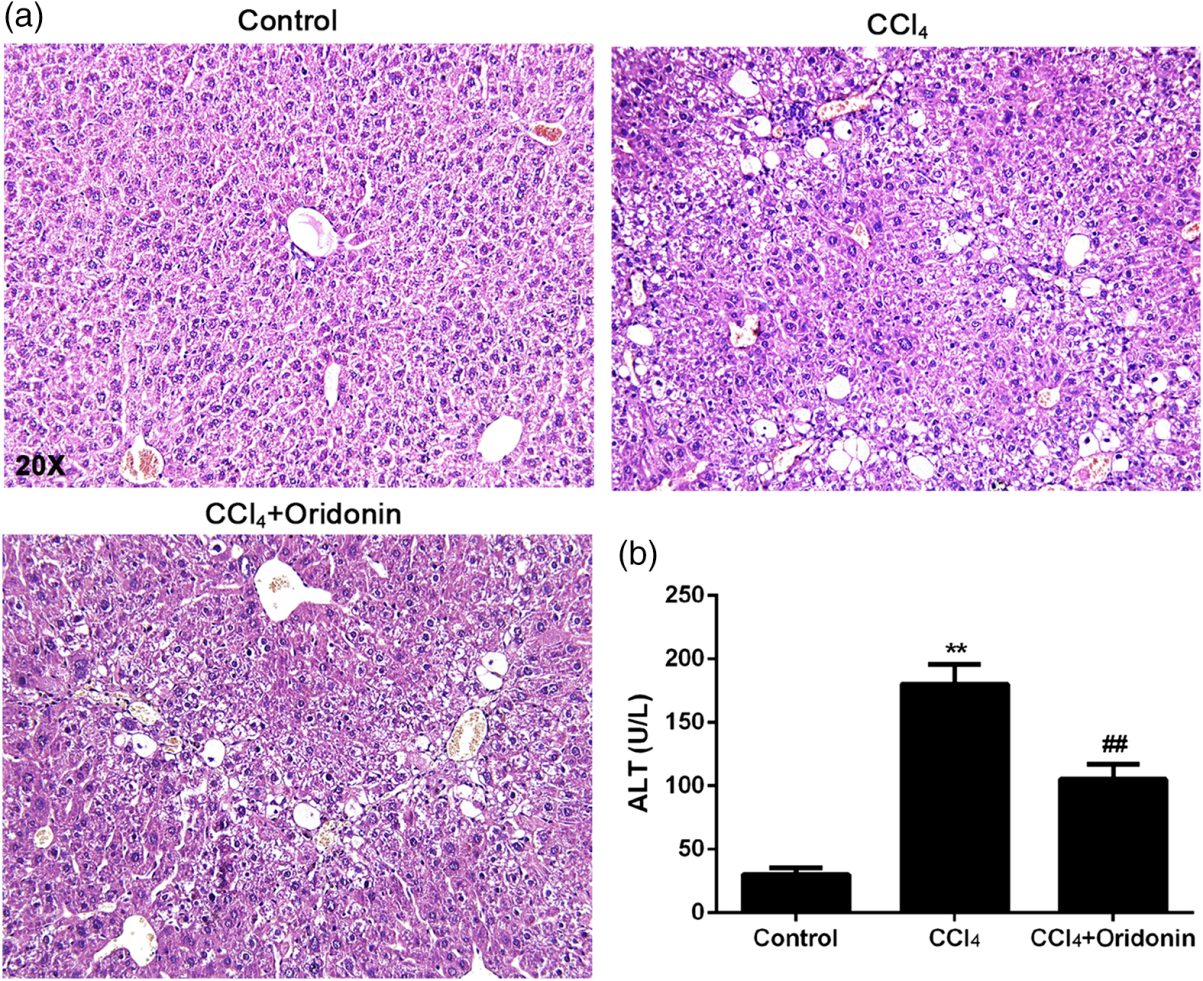
The effect of oridonin on CCl_4_‐induced liver injury in mice. Representative histological images of H&E stained liver sections (a) and serum ALT (b) of control mice, CCl_4_‐injected mice and CCl_4_‐injected mice treated with oridonin (5 mg/kg. i.p.). **p < .01 versus control group, ## *p* < .01 versus CCl_4_‐injected group, Original magnification 20×, CCl_4_, carbon tetrachloride

H&E staining of CCl_4_‐induced mouse livers showed severe damage. The observed elevation of serum ALT level also indicated liver injury. In contrast, oridonin treatment for 6 weeks significantly attenuated liver damage (Figure 2a). Figure 2b showed that the administration of oridonin also significantly reduced the elevated ALT in mice.

### 3.2 Oridonin decreased the deposition of collagen in CCl_4_‐induced liver fibrosis

**Figure 3:**
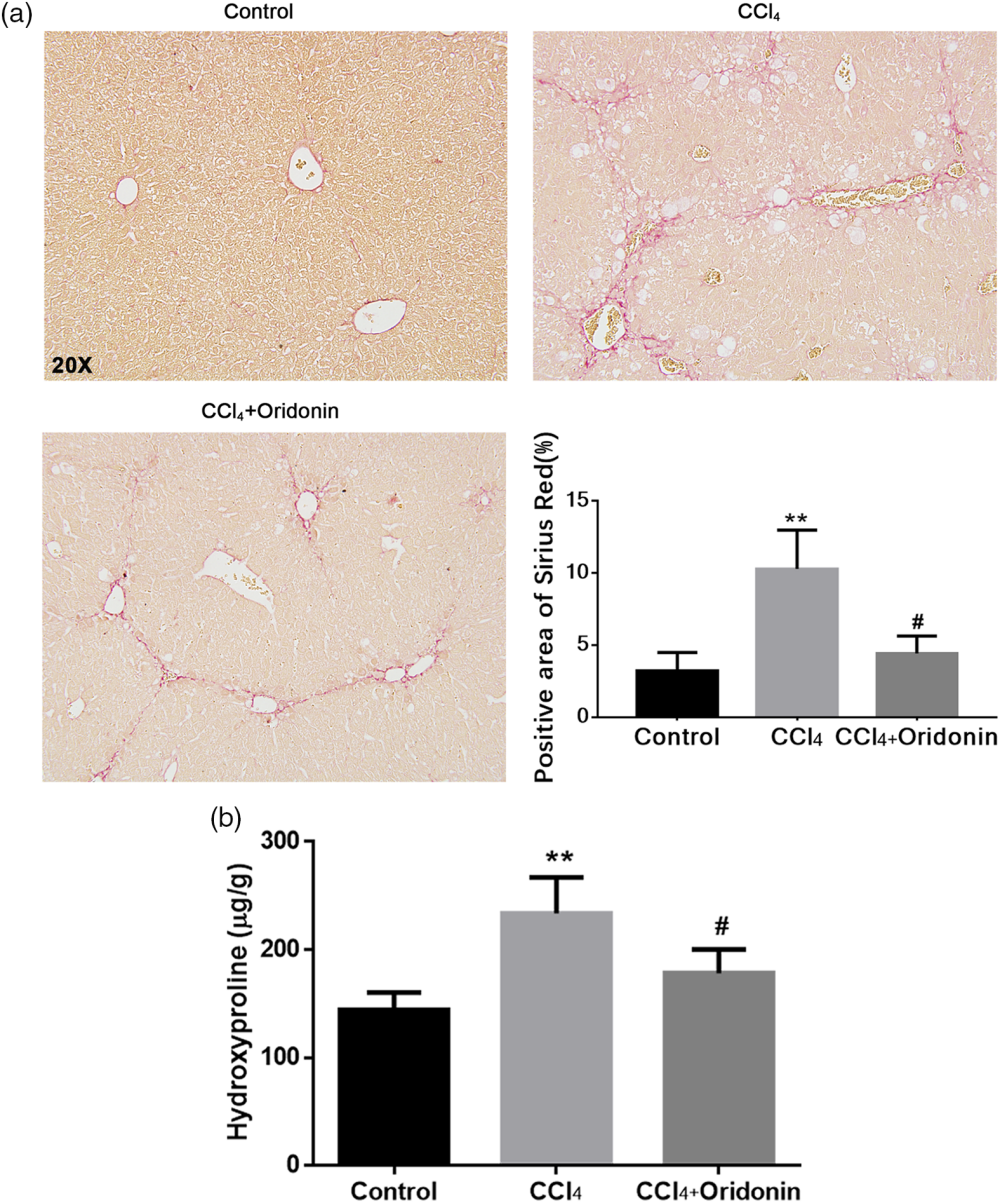
The effect of oridonin on the deposition of collagen in CCl_4_‐induced liver fibrosis. Representative histological images of Sirius Red staining of liver sections (a) and the level of hydroxyproline (b) of control mice, CCl_4_‐injected mice and CCl_4_‐injected mice treated with oridonin (5 mg/kg. i.p.). ** *p* < .01 versus control group, #*p* < .05 versus CCl_4_‐injected group, Original magnification 20×

Sirius Red staining of CCl_4_‐induced mouse livers showed marked collagen deposition, whereas there was less collagen deposition in the livers of mice treated with oridonin (Figure 3a). In addition, the level of hydroxyproline, a characteristic amino acid of collagen, was significantly decreased in the livers of oridonin‐treated mice in contrast to CCl_4_‐induced fibrotic livers without treatment (Figure 3b).

### 3.3 Oridonin inhibited the activation of HSCs in experimental murine liver fibrosis

**Figure 4:**
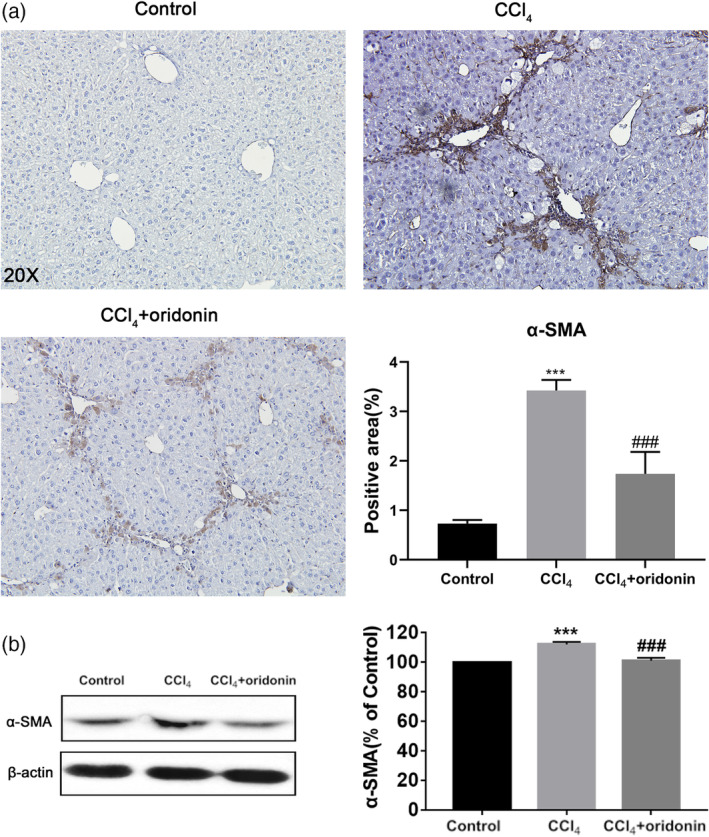
Oridonin inhibited the activation of HSCs in vivo. Representative immunohistochemical staining images of α‐SMA of liver sections (a) and the protein expression and densitometric analysis of α‐SMA (b) of control mice, CCl_4_‐injected mice and CCl_4_‐injected mice treated with oridonin (5 mg/kg. i.p.). *** *p* < .001 versus control group, ###*p* < .001 versus CCl_4_‐injected group, Original magnification 20×; α‐SMA, α‐smooth muscle actin

The expression of α‐SMA, a marker for activated HSCs, was examined by immunohistochemistry and western blotting. Immunohistochemical assay results demonstrated that the expression of α‐SMA was notably higher in the liver tissues of the CCl_4_‐intoxicated group. Compared with the CCl_4_ model group, the α‐SMA‐positive areas in mice treated with oridonin were significantly smaller (Figure 4a). In concordance with immunohistochemical results, immunoblotting assays showed that the expression of α‐SMA was increased in livers with CCl_4_‐induced fibrosis. However, the expression of α‐SMA was significantly repressed in the livers of mice treated with oridonin (Figure 4b). These results suggest that oridonin can efficiently attenuate the activation of HSCs.

### 3.4 Downregulation of NLRP3 inflammasome by oridonin prevented the activation of HSCs

**Figure 5:**
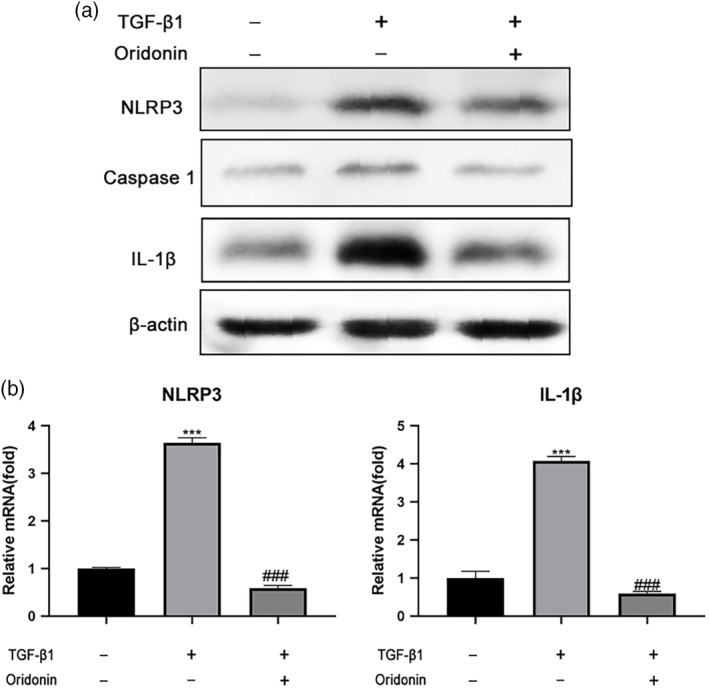
Oridonin regulates the NLRP3 inflammasome in TGFβ1‐stimulated LX‐2 cells. Representative western blotting analysis of protein expression (a) of NLRP3, caspase‐1, IL‐1β, and qPCR analysis of mRNA level (b) of NLRP3, IL‐1β following TGFβ1 (2 ng/ml) stimulation and oridonin (2.5 μM) treatment. β‐Actin serves as an internal control, *** *p* < .001 versus control group, ###*p* < .001 versus CCl_4_‐injected group

Oridonin can inhibit the NLRP3 inflammasome which plays a key role in the activation of HSCs. TGFβ1 was used to induce the activation of a human HSCs cell line, LX‐2. Compared to cells without TGFβ1 treatment, the expression of NLRP3 was markedly increased in mice treated with TGFβ1. The increase was blocked by oridonin treatment. Caspase‐1 protein level was increased after NLRP3 inflammasome activation. Caspase‐1 shears pro‐IL‐1β into mature IL‐1β to promote inflammation and fibrosis. Similar to NLRP3, the protein expression of caspase‐1 and IL‐1β was upregulated with TGFβ1 treatment and downregulated in the oridonin treatment group (Figure 5).

### 3.5 Oridonin reduced the recruitment of Kupffer cells

**Figure 6:**
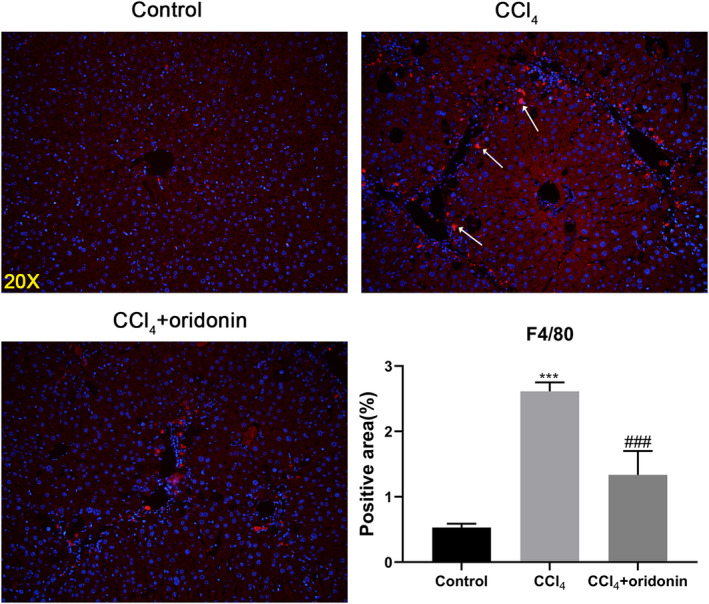
Oridonin reduced the number of liver Kupffer cells. Representative immunofluorescence staining images of F4/80 of liver sections of control mice, CCl_4_‐injected mice and CCl_4_‐injected mice treated with oridonin (5 mg/kg. i.p.). *** *p* < .001 versus control group, ###*p* < .001 versus CCl_4_‐injected group, Original magnification 20×

We next investigated the anti‐inflammatory effect of oridonin on inflammatory cells in the liver such as the Kupffer cells. As expected, compared with the control group, the expression of F4/80, a marker of Kupffer cells, was increased in the CCl_4_‐treated group. In contrast to the CCl_4_‐treated group, the expression of F4/80 was markedly decreased in mice treated with oridonin. This suggested that oridonin can reduce the recruitment of Kupffer cells and their accompanying inflammatory effects (Figure 6).

## 4 DISCUSSION

Liver fibrosis is still an intractable disease today without effective treatment options due to its intricate pathogenesis (Lin et al., 2018). In response to liver injury, HSCs are activated and transdifferentiated into myofibroblasts that produce proinflammatory cytokines. The accumulation of ECM is the immediate cause of liver fibrosis. ECM can, in turn, modulate the activation and proliferation of HSCs (Hernandezgea & Friedman, 2011). Thus, decreasing ECM deposition is a pivotal strategy for the treatment of liver fibrosis.

Oridonin is one of the multitudinous drug candidates for the treatment of liver fibrosis. It is extracted from *Rabdosia rubescens* which is an herbal medicine. Oridonin has been reported to be an effective anti‐inflammatory molecule in the treatment of gouty arthritis, type 2 diabetes, and peritonitis and it functions by inhibiting NLRP3 activation (He et al., 2018). It is also a potential compound for leukemia treatment and shows curative effects for liver cancers (H. Wang et al., 2011; Zhen et al., 2012; Zhou, Chen, Wang, & Chen, 2007). In addition, Kuo et al. (2014) found that oridonin can potentially be used to treat liver fibrosis by decreasing intracellular GSH concentration and increasing ROS formation and caspase activation to cause apoptosis in HSCs. However, the in vivo activities of oridonin are unclear. Our current study demonstrated that oridonin is a potential candidate for the treatment of chronic liver fibrosis as it inhibits collagen deposition in mice.

Specifically, in this study, H&E staining results showed that the intraperitoneal injection of CCl_4_ for 6 weeks caused severe damage and inflammatory cell infiltration in mice livers. However, these effects were ameliorated after administrating oridonin for 6 weeks. This result supports the findings of Zhao and colleagues which stated that oridonin plays an anti‐inflammatory role by decreasing the production of proinflammatory cytokines (G. Zhao, Zhang, et al., 2017). Furthermore, the level of ALT was significantly increased in mice after CCl_4_ injection compared to the control group. The observed decrease in ALT level after treatment with oridonin suggests that oridonin has hepatoprotective effects. Deng et al. (2017) suggested that oridonin exhibits hepatoprotective effects by inhibiting apoptosis in hepatocytes. Furthermore, the ability of oridonin to increase the expression and activity level of a hepatic drug‐metabolizing enzyme (CYP450) was verified. Oridonin might thus work in synergy with other hepatoprotective drugs (X. Dong, Liu, & Li, 2015; T. Xu, Jin, Wu, Ye, & Li, 2017; Y. W. Zhang, Bao, Hu, Qu, & Zhou, 2014).

Oridonin and its analog CYD0682 could also downregulate α‐SMA and ECM protein levels in HSCs (Bohanon et al., 2014; Bohanon, Wang, Graham, Ding, et al., 2015). Our in vivo test results confirmed this mechanism. The content of hydroxyproline, a characteristic amino acid in collagen, increased with the progression of liver fibrosis. This increase was remarkably inhibited by oridonin administration. In concordance with the hydroxyproline assay results, Sirius Red staining results showed that the collagen fibers in the mouse liver were reduced by oridonin treatment. This suggests that oridonin ameliorated liver fibrosis by inhibiting collagen deposition. Western blot assay showed that the protein expression of α‐SMA, which is a marker of activated HSCs, was significantly increased in livers with CCl_4_‐induced fibrosis. However, the expression of α‐SMA was significantly suppressed in the liver sections of mice treated with oridonin. It is worth noting that many other natural compounds have been shown to anti‐inflammation and anti‐fibrosis, such as dioscin (Gu et al., 2016; X. Zhang et al., 2015) and Emodin (M. X. Dong et al., 2009; Gui et al., 2007), but there were a few compounds targeted specifically at NLRP3, our study has demonstrated that drug inhibiting NLRP3 could exert good anti‐fibrotic effect for the first time.

In conclusion, our current study demonstrates that oridonin inhibits collagen deposition and inflammation to attenuate CCl_4_‐induced liver injury and fibrosis in mice. Nevertheless, the experiments carried out in this study only involved a narrow and superficial range of pharmacological identifications. Thus, further study should be performed to obtain a clearer picture of the liver‐protective activities of oridonin. These results should also be compared with the current first‐line treatment drugs for liver fibrosis.

## CONFLICT OF INTEREST

The authors declare no conflict of interest.
